# Gender Differences in Preclinical Markers of Kidney Injury in a Rural North Carolina African-American Cohort

**DOI:** 10.3389/fpubh.2015.00007

**Published:** 2015-01-26

**Authors:** Mildred A. Pointer, Kianda Hicks, ClarLynda Williams-Devane, Candace Wells, Natasha Greene

**Affiliations:** ^1^Julius L. Chambers Biomedical/Biotechnology Research Institute, North Carolina Central University, Durham, NC, USA; ^2^Department of Biology, North Carolina Central University, Durham, NC, USA; ^3^Department of Nutrition, North Carolina Central University, Durham, NC, USA; ^4^Department of Nursing, North Carolina Central University, Durham, NC, USA

**Keywords:** diabetes, hemoglobin A1c, African-American, gender, sex

## Abstract

**Introduction:** The incidence rate of end-stage renal disease (ESRD) is highest among African-American (AA) males. The reason for this disparity in ESRD for AA males remains unclear, but it is well established that diabetes is the leading risk factor. Prediabetes may also be a risk for kidney disease since prediabetics have increased risk for cardiovascular disease and often do not receive drug interventions unless their hemoglobin A1c (A1c) level is above 6%. Perhaps, AA males are at greater risk because they often are untreated prediabetics and this predisposes them to renal injury. Therefore, we hypothesize that prediabetic AA males have higher albumin:creatinine ratio (ACr), a biomarker of renal injury, than their female counterparts.

**Methods:** Male and female AAs were recruited (53 females and 47 males; 45 ± 2 years old) from a rural northeastern region of NC. Blood and urine samples were collected for A1c and albumin measurements, respectively. Participants were stratified based on their A1c levels: non-diabetic: <5.7%, prediabetic: ≥5.7% but <6.5%, and diabetic: ≥6.5%.

**Results:** The proportion of males that are normal, prediabetic, and diabetic differed from that of females (*p* = 0.002). Interestingly, prediabetic men tended to be younger (41 ± 4 vs. 51 ± 3, respectively; *p* = 0.027) than prediabetic females (*p* = 0.027). A1c and ACr were not associated with blood pressure in males or females. AA males had a relative risk of 0.9, 2.5, and 1.4 for microalbuminuria for non-diabetic, prediabetic, and diabetic, respectively, compared to AA females.

**Conclusion:** These results support our hypothesis that AA males may be predisposed to prediabetes kidney injury compared to their female counterpart. Thus, young AA males should be screened for biomarkers of kidney injury even if they have normal glucose and blood pressure levels.

## Introduction

A recent report on premature deaths in North Carolina reveals that the leading causes of death among non-whites include diabetes, kidney disease, stroke, and heart disease (1). The cause(s) of the disproportionate premature deaths due to diabetes and kidney disease in non-whites is of particular interest for two reasons. First, the two diseases are intricately linked in that diabetes is the leading risk factor for end-stage renal disease (ESRD) (2). Second, ESRD, the last stage individuals experience prior to complete kidney failure, is a major public health concern with a mortality rate that exceeds that for cancer and heart attacks (241/1,000 patient years at risk compared to 137/1,000 and 116/1,000 for cancer and heart attacks, respectively) (2).

African-Americans (AAs) have a fourfold greater risk for ESRD compared to whites (2). Although AAs make up approximately 12% of the general population, they make up more than one-third of the dialysis patents (3). It is of particular interest that there is a gender difference in kidney disease among AA population; AA males have a 2.4-fold greater risk of developing kidney disease compared to AA females (4) and have renal disease at a younger age (5). In this study, we addressed the greater predisposition of AA males toward kidney disease as compared to that of AA females. Given that diabetes is the leading risk factor for ESRD, kidneys, therefore, appear to be particularly prone to injury during chronic hyperglycemia. For AA males, kidneys may be predisposed to injury during lower glycemic levels normally not recognized as diabetic, i.e., renal injury may very likely occur during prediabetes. Therefore, the objectives for this study were to determine whether (1) relative proportion of AA males that are normal, prediabetic, and diabetic differs from that found in females; (2) prediabetic males are younger than prediabetic females; (3) A1c is correlated with age; and (4) relative risk for microalbuminuria is greater in prediabetic and diabetic males compared to female counterparts.

To investigate this possibility, we recruited AA males and females from a region of NC with high rates of diabetes and kidney disease (6, 7) and measured their hemoglobin A1c levels for stratification into non-diabetes, prediabetes, and diabetes groups. Because blood pressure is a known risk factor for kidney disease, we also measured systolic and diastolic blood pressure (DBP).

## Materials and Methods

### Participants

Adults of African descent (100; 57 females, 43 males) were recruited to participate in the cross-sectional Calcium Homeostasis and Metabolic Problems Study (CHAMPS; Mildred A. Pointer, PI) from the Halifax County region of North Carolina. Participants were volunteers recruited during a health fair held at one of the local churches in the area as part of the *Diabetes Family Project* (Dr. Natasha Greene) recruitment activities. All study procedures and materials were approved by, and in compliance with, the North Carolina Central University institutional review board. Eligibility criteria for entry were: (1) being 18–80 years old and (2) living in the region. This age range was selected to capture individuals who could be included in the non-diabetic, prediabetic, and diabetic strata. Type 2 diabetes usually manifests in relatively older individuals while younger individuals are usually found to be prediabetic or non-diabetic (8). Exclusion criteria included being under the age of 18 years old and unable to understand English.

### Study protocol

All participants were consented before any data were collected. Each participant was allowed to rest for 5 min before resting blood pressures were measured. Once blood pressure was taken, a short survey with questions about their socioeconomic background was administered and blood and urine samples were collected. Each participant received a small remuneration once they had completed the entire protocol.

### Blood pressure measurements

After receiving informed consent, trained staff measured blood pressure by the sphygmomanometer method with a GE Dinamap Pro 100 automatic model and a cuff size appropriate for the body size. Each participant was allowed 5 min to sit quietly before taking the first resting baseline parameters. The Dinamap was set to measure systolic blood pressure (SBP) and DBP at 1-min intervals for 5 min for the baseline resting measurement. Resting baseline blood pressures were calculated as the average of the three readings.

### Plasma and urine samples

Blood samples were collected by LabCorp phlebotomists and North Carolina Central University nursing students for measurement of hemoglobin A1c, BUN, and creatinine. Plasma was isolated onsite and stored in ice filled coolers. A spot urine was collected for the measurement of total volume, albumin, protein, and creatinine. All samples (plasma and urine) were delivered to LabCorp on the same day of the event and LabCorp performed all blood and urine assay measurements.

### Stratification using preclinical markers

Participants were classified as normal, prediabetic, and diabetic based on established A1c marker cutoffs as recommended by the American Diabetes Association (9). Specifically, non-diabetics were defined as having A1c levels that were <5.7%, prediabetic as A1c levels that were ≥5.7% but <6.5%, and diabetic as having A1c levels that were ≥6.5%. Microalbuminuria in spot urine (10–12) was determined using sex-specific albumin:creatinine ratio (ACr) cut-points as previously published (11). We used ≥17 μg/mg creatinine for males and ≥25 μg/mg for females (11).

### Data analysis

We used Chi-Square analysis to determine whether the relative proportion of AA males that are normal, prediabetic, and diabetic differs from the proportion found in AA females. Student’s *t*-test was used to determine whether prediabetic males are younger than prediabetic females and Pearson product–moment correlation analysis determined whether A1c is correlated with age. Values are expressed as mean ± SEM.

## Results

Table 1 describes our sample demographics. In this sample population of AAs from rural Halifax County in North Carolina, we had 53 females and 47 men. The females had higher hemoglobin A1c levels (6.4 ± 0.2 vs. 5.8 ± 0.3; *p* < 0.001) and were older than the men (49 ± 2 vs. 41 ± 2 years; *p* < 0.011). Systolic and DBPs as well as fasting glucose levels were not significantly different between men and women. There were no significant differences between males and females in terms of level of education and income. However, there was a trend for males to have a lower level of education (*p* = 0.07).

**Table 1:** **Sample population characteristics**.

Characteristic	Whole group, mean ± SE (*n*)	Women, mean ± SE (*n*)	Men, mean ± SE (*n*)	*P* (women vs. men)
Age	45 ± 2 (100)	49 ± 2 (53)	41 ± 2 (47)	**0.011**
HA1c (%)	6.1 ± 0.2 (94)	6.4 ± 0.2 (50)	5.8 ± 0.3 (44)	**<0.001**
SBP (mmHg)	137 ± 2 (100)	136 ± 3 (53)	138 ± 3 (47)	0.576
DBP (mmHg)	77 ± 1 (100)	75 ± 3 (53)	79 ± 2 (47)	0.062
Glucose (mg/dL)	95 ± 4 (98)	94 ± 3 (51)	96 ± 8 (47)	0.657
Income (%)				
<$20,000	66	58	74	0.07
$20,000–$50,000	29	33	26	
>$50,000	5	9	0	
Education (%)				
Less than HS	29	27	33	0.205
HS/GED	55	52	58	
Bachelor	11	11	9	
Post-bac	5	10	0	

*Values = mean ± SE; ranked sum *t*-test, Chi-Square comparison between males and females. The bold indicates statistically significant*.

All participants were categorized as non-diabetic, prediabetic, and diabetic using the A1c levels. Those with A1c levels <5.7% were classified as non-diabetic; those with A1c values ≥5.7 but <6.5% were assigned to the prediabetic group; and those with A1c ≥6.5% were assigned to the diabetic group. Because we were unable to get A1c values measured in six participants, these were not included in the analyses. As shown in Table 2, the frequency distribution differed between males and females (*p* < 0.03). Among males, the lowest proportion of males was in the diabetic group whereas among the females, the lowest proportion was found in the normal classification. Thus, more females were found within the prediabetic and diabetic classification (78%) while more of the males were within the normal and prediabetic classification (91%). Although the mean age for the non-diabetic and diabetic groups did not differ between the sexes, the prediabetic males were younger than females (41 ± 4 vs. 51 ± 3, respectively; *p* = 0.027).

**Table 2:** **Gender differences in classification based on hemoglobin A1c levels**.

	Males				Females*			
	*n*	Age	%	A1c	*n*	Age	%	A1c
Normal	24	36 ± 5	55	5.3 ± 0.05	11	30 ± 4	22	5.3 ± 0.05
Prediabetic	16	41 ± 4**	36	5.9 ± 0.03	23	51 ± 3	46	6.0 ± 0.04
Diabetic	4	63 ± 6	9	8.9 ± 1.5	16	58 ± 2	32	7.6 ± 0.52

*Chi-squared normal, prediabetes, diabetes proportion analysis; p < 0.003 compared to males.

**p = 0.027, t-test between male and females; values = mean ± SE.

**Figure 1:**
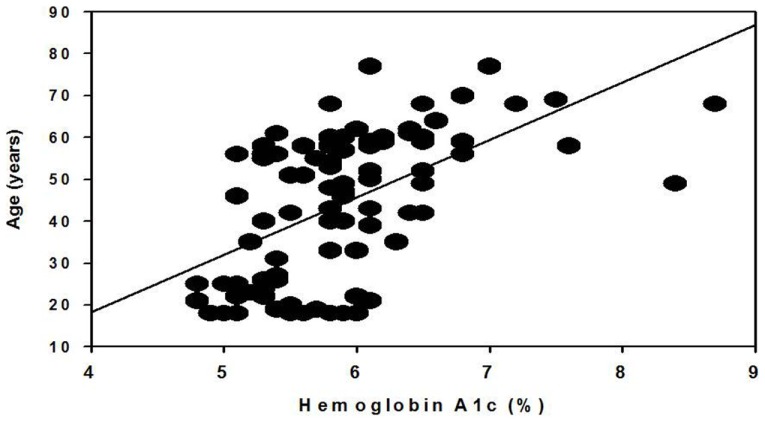
**Correlation of hemoglobin A1c and age**. Data include both male and female participants from a rural North Carolina cohort of the African-American Calcium Homeostasis and Metabolic Problems Study (Mildred A. Pointer, PI).

Because we observed a difference in the age between prediabetic males and females, we performed a Pearson product–moment correlation between age and A1c for the entire cohort and for males and females separately. As shown in Figure 1, there is a significant correlation between age and A1c, specifically, older individuals have higher A1c levels (*p* = 0.0000000838). This relationship was independent of gender (males, *p* = 0.0025; and females, *p* = 0.00000348).

Our primary interest was whether males were at a greater risk for renal injury even with lower glycemic levels. We identified males and females with microalbuminuria using standard definitions of microalbuminuria by gender (urinary albumin:creatinine levels between 17 and 250 μg/mg for males and 25–355 μg/mg for females). Using sex-specific albumin:creatinine cut off instead of the often used clinical set point of ≥30 μg/mg was preferred because the clinical set point appears to underestimate microalbuminuria for men and AAs (11). Because of the tendency for males to have higher creatinine levels, we found that males had lower ACr compared to females (data not shown).

As shown in Table 3, among males the percentage with ACr values above the recommended normal ACr levels was 9, 31.25, and 50% in the non-diabetic, prediabetic, and diabetic groups, respectively. We observed lower frequencies of microalbuminuria of 10, 12.5, and 35.7% in the non-diabetic, and diabetic groups, respectively in the females. Consequently, the relative risk for microalbuminuria for males compared to females is 0.9, 2.5, and 1.4 in non-diabetic, prediabetic, and diabetics, respectively. In this cohort, prediabetic males appear to be predisposed to renal injury relative to females.

**Table 3:** **Frequency of microalbuminuria in a rural cohort of African-American males and females**.

	Non-diabetic (%)	Prediabetes (%)	Diabetes (%)
Males	9	31.25	50
Females	10	12.5	35.7
Relative risk^a^	0.9	2.5	1.4

*^a^Comparing males to females*.

## Discussion

We show in this report that rural AA males living in the Halifax County of North Carolina differ from their female counterpart in the following ways: (1) the proportion of males who were normal, prediabetic, and diabetic differed from that of females; (2) prediabetic males are younger; and (3) prediabetic males are at greater risk for renal injury. We interpret these findings to mean that AA males are predisposed to kidney disease because: (1) exposure to prediabetes status (a status that often does not include drug treatment) at a younger age may ultimately mean longer exposure to an injury-promoting stimulus; and (2) their kidneys may be more sensitive to the injury caused by hyperglycemia. This report suggests new areas of investigation that could result in new insights into why AA males are at greater risk for kidney disease.

Currently, prediabetes status means that you are at greater risk for diabetes and that lifestyle modification should be implemented to reduce that risk; however, medications are usually not prescribed unless the A1c levels are above 6% (13). For every diabetic identified, there are approximately four prediabetics. Prediabetics are threefold to sevenfold more likely to develop diabetes than non-diabetics. Interestingly, prediabetes has its own added risk for cardiovascular disease (30–60% higher risk). The good news is that lifestyle modification can prevent and delay progression to frank diabetes (13, 14). There are also a limited number of studies in which the use of diabetes drugs such as rosiglitazone can reduce the incidence of type 2 diabetes as well as increase the regression to normoglycemia in prediabetes (15). Thus, it is reasonable to expect that treatment of young AAs males with prediabetes could significantly reduce their progression toward diabetes, but more than that, could prevent or delay the adverse complications of diabetes such as kidney disease, retinopathy, and neuropathy.

Because diabetes is the leading risk factor for kidney disease, we also examined whether microalbuminuria was highest in those with higher A1c levels. Others have observed in an AA cohort of over 1,000 patients that higher ACr is seen in those with higher A1c levels (16). We were not able to confirm this association with our smaller sample size; however, we did observe that microalbuminuria frequency was higher in prediabetics and diabetics in both males and females compared to non-diabetics (Table 3). Nearly one-third of the prediabetic males had microalbuminuria. Compared to females, they were 2.5 times more likely to have microalbuminuria despite similar levels of A1c. Although we hypothesized that males would have higher (ACr) compared to females, we did not find this to be the case. This is likely due to the tendency for males to have higher creatinine excretion (11). Nonetheless, the overall hypothesis of greater risk for renal injury in males was supported by our frequency of microalbuminuria results.

It is unclear why there is a difference in A1c association with renal injury between males and females. One interpretation of the results is that males have a lower threshold value of A1c before overt signs of renal injury begins to be exhibited. In other words, males show a more sensitive association of A1c levels with microalbuminuria. The challenge for AA males is that a urinary analysis may not be requested since their A1c levels are not considered diabetic. By the time their A1c levels are elevated to diabetes status, the renal injury may have progressed to early stages of chronic kidney disease. Thus, it may be important for health care professionals to provide male consultations about changes in lifestyle to prevent progression to ESRD and to provide treatment interventions that will reduce plasma glucose levels. We recognize that our sample size is relatively small and that more studies are warranted to confirm this difference between AA males and females; however, these preliminary findings may highlight an area requiring immediate attention if we are to address this major health issue of disparate kidney disease prevalence in AA males.

Interestingly, the participants were not hypertensives. Furthermore, blood pressure was not different among the different stratifications for males or females. Consequently, the microalbuminuria observed was independent of blood pressure (Table S1 in Supplementary Material).

Other factors that could explain the increased risk for renal injury in AA males include psychosocial factors. For example, socioeconomic status has been reported to be a significant contributor to kidney disease, particularly in AAs (17, 18). After adjusting for demographics, socioeconomic status was a risk for kidney disease in AAs only (18). Stress is another factor that could both contribute to kidney disease as well as explain the increased risk in males. We have shown that physiological stress can exacerbate the effect of high salt on kidney injury in an animal model of hypertension that resembles that observed in AAs (19, 20). While dietary salt can increase kidney injury apart from hypertension, stress can aggravate this effect of salt on kidney injury (20). Moreover, we have shown that the experience of racism is a significant determinant of resting blood pressure in young healthy AAs (21). Consequently, environmental stress such as racism, dietary salt, and financial strain could contribute to increased risk for kidney disease and may also explain the gender differences. Studies reveal that the AA male is more likely to experience discrimination as compared to the AA female and this could explain the higher unemployment rate among AA males (22). Taken together, AA males may be more predisposed to renal injury despite lower glucose levels due to the added stress of being AA male. Further studies are required to determine whether stress plays a role in the increased risk for renal injury during prediabetes.

### Study limitations

The obvious limitation of this study is the small cross-sectional sample size. Additionally, we sampled from a single region within the northeastern part of North Carolina. Consequently, our findings must be reproduced in a larger sample and from diverse regions before they can be applied to AAs in general.

Another limitation is that the amount of information collected at the time of data collection. In the past, we have observed that participants from community locations as opposed to participants who come to our University location become impatient if the time for the protocol completion went beyond about 30 min or if they had to answer a long survey of questions. For this reason, our protocol and surveys are designed to be completed in about 20–30 min when we visit community sites. We attempted to obtain medications but many could not provide the names of the medications. Other information that was not collected but may be helpful in uncovering the reasons for the gender differences include: whether participants have a primary care physician; and whether participants have had a complete physical examination within the past 2 years and what tests were performed.

Failure to do repeat sampling is also another concern of this study. However, our intent was to identify potential new areas of research focus to address the major health concern of ESRD, particularly in AA males.

### Perspectives

End-stage kidney disease is a major health concern. There are limited treatment options and the options that are currently available cost $50 billion annually (Medicare and non-Medicare; (2). This cost is even greater if lost wages due to illness and treatment are considered (23). In addition to the economic burden of ESRD, there is an accompanying psychosocial burden that includes poor quality of life and strained social relationships (23). Consequently, if we can identify new areas for future research that can provide new insight into the underlying cause of kidney injury, particularly among AAs, we can make significant strides in reducing this health and economic burden.

Previous findings reveal that prediabetics are threefold to sevenfold more likely to develop diabetes than non-diabetics and, interestingly, prediabetes has its own added risk for cardiovascular disease (30–60% higher risk) (14, 24). Therefore, prediabetes may also be an added risk for renal disease. One area where we may be able to make a significant impact is in the area of standard of care for prediabetes in AA. The current standard of care for prediabetics is to inform individuals of their increased risk, give counseling on lifestyle changes, and initiate drug interventions only if A1c levels are above 6% (25). If prediabetes does indeed convey underlying risk for disease development, then failure to treat prediabetes may actually be allowing the renal injury to advance.

In this study, we show important differences between AA males and females that could help to explain the proclivity of AA males for kidney disease. These differences include: (1) more males are prediabetic; (2) prediabetic males are younger; and (3) prediabetic males are at greater risk for microalbuminuria. The combination of these differences could result in longer duration of prediabetes without diabetic drug intervention and this prolonged exposure to prediabetes without more aggressive interventions may enable the quiet storm of underlying diabetes complications leading to kidney disease. Surprisingly, blood pressure did not appear to be a risk for microalbuminuria in either males or females. If we are to address the major health concern of ESRD, it may be important for health care professionals to be particularly vigilant about beginning drug interventions along with the consultations for young AA males even though they may not be diagnosed as having frank diabetes.

Although there are limitations to this study as discussed above, our results raise some intriguing questions. Making significant progress in reducing the risk for kidney disease and ultimately the morbidity and mortality associated with this disease, we may need to turn our efforts to finding answers to such questions as: (1) what is the underlying pathophysiology of kidney disease that occurs during prediabetes; (2) does the renal pathophysiology associated with prediabetes differ between males and females; and (3) what other factors unique to AA males may contribute to renal injury apart from hypertension and diabetes. The answers to such questions may lead to the development of new treatment interventions that are more personalized for and, consequently, more effective in mitigating the progression to ESRD.

## Conflict of Interest Statement

The authors declare that the research was conducted in the absence of any commercial or financial relationships that could be construed as a potential conflict of interest.

## Supplementary Material

The Supplementary Material for this article can be found online at http://www.frontiersin.org/Journal/10.3389/fpubh.2015.00007/abstract
