# Supplementary material for: Gender Differences in Preclinical Markers of Kidney Injury in a Rural North Carolina African-American Cohort
Source: Front Public Health. 2015 Jan 26;3:7. doi: 10.3389/fpubh.2015.00007 (PMC4306298; doi:10.3389/fpubh.2015.00007)
Supplement: Supplementary file 1 [file DataSheet_1.DOCX]

Supplemental Table 1. *Blood Pressure Levels in a Rural African American Male and*

*Female Cohort.*

|  | **Males** | | **Females** | |
| --- | --- | --- | --- | --- |
|  | **SBP** | **DBP** | **SBP** | **DBP** |
| **Normal** | 137±4 | 79±3 | 130±8 | 72±5 |
| **Pre-diabetic** | 135±3 | 79±2 | 139±4 | 78±2 |
| **Diabetic** | 134±4 | 82±4 | 135±5 | 73±3 |
